# Convergent Epigenetic Mechanisms Avoid Constitutive Expression of Immune Receptor Gene Subsets

**DOI:** 10.3389/fpls.2021.703667

**Published:** 2021-09-07

**Authors:** Damián Alejandro Cambiagno, José Roberto Torres, María Elena Alvarez

**Affiliations:** ^1^Unidad de Estudios Agropecuarios (UDEA), INTA-CONICET, Córdoba, Argentina; ^2^Departamento de Química Biológica Ranwel Caputto, Facultad de Ciencias Químicas, Centro de Investigaciones en Química Biológica de Córdoba, CIQUIBIC, CONICET, Universidad Nacional de Córdoba, Córdoba, Argentina

**Keywords:** *PRR*/*NLR* immune receptor genes, epigenetics, 5-mC/H3K9me2 and H2A.Z/H3K27me3 marks, defense cascades, priming

## Abstract

The gene pool encoding PRR and NLR immune receptors determines the ability of a plant to resist microbial infections. Basal expression of these genes is prevented by diverse mechanisms since their hyperactivity can be harmful. To approach the study of epigenetic control of *PRR*/*NLR* genes we here analyzed their expression in mutants carrying abnormal repressive 5-methyl cytosine (5-mC) and histone 3 lysine 9 dimethylation (H3K9me2) marks, due to lack of MET1, CMT3, MOM1, SUVH4/5/6, or DDM1. At optimal growth conditions, none of the mutants showed basal expression of the defense gene marker *PR1*, but all of them had greater resistance to *Pseudomonas syringae* pv. *tomato* than wild type plants, suggesting they are primed to stimulate immune cascades. Consistently, analysis of available transcriptomes indicated that all mutants showed activation of particular *PRR/NLR* genes under some growth conditions. Under low defense activation, 37 *PRR*/*NLR* genes were expressed in these plants, but 29 of them were exclusively activated in specific mutants, indicating that MET1, CMT3, MOM1, SUVH4/5/6, and DDM1 mediate basal repression of different subsets of genes. Some epigenetic marks present at promoters, but not gene bodies, could explain the activation of these genes in the mutants. As expected, *suvh4/5/6* and *ddm1* activated genes carrying 5-mC and H3K9me2 marks in wild type plants. Surprisingly, all mutants expressed genes harboring promoter H2A.Z/H3K27me3 marks likely affected by the chromatin remodeler PIE1 and the histone demethylase REF6, respectively. Therefore, MET1, CMT3, MOM1, SUVH4/5/6, and DDM1, together with REF6, seemingly contribute to the establishment of chromatin states that prevent constitutive *PRR/NLR* gene activation, but facilitate their priming by modulating epigenetic marks at their promoters.

## Introduction

Plant genomes encode for large families of immune receptor proteins that perceive the presence of pathogens and consequently activate defenses. Pattern recognition receptors (PRR) detect microbe-associated molecular patterns (MAMPs) at the cell surface, while intracellular nucleotide-binding leucine-rich repeat (LRR) proteins (NLR) recognize pathogen effectors within the cell. PRR/NLR receptors are master regulators of immune cascades that affect the expression of thousands of defense genes (Block and Alfano, 2011; Macho and Zipfel, 2014). Their activity is regulated at transcriptional and post-transcriptional levels by convergent mechanisms that maximize defenses with low fitness costs, since overstimulation of immune responses can lead to reduced growth or seed production, or cause cell death (Karasov et al., 2017). *NLR/PRR* genes can increase their expression (Dowen et al., 2012; Yu et al., 2013), or alter their alternative splicing or polyadenylation (Tsuchiya and Eulgem, 2013; Lai et al., 2020) in response to pathogens or elicitors, and both processes are affected by chromatin epigenetic marks. *NLR* gene clusters are enriched in transposable elements (TEs) that concentrate repressive marks, such as 5-methylcytosine (5-mC) and di-methylation of histone H3 at lysine 9 (H3K9me2). High resolution DNA methylation profiling showed that induction of some *PRR/NLR* genes correlates with loss of 5-mC from TEs harbored within or near these loci (Dowen et al., 2012; Yu et al., 2013). Moreover, the epigenetic marks of TEs could also affect *PRR/NLR* expression in *trans* (Pavet et al., 2006; Cambiagno et al., 2018). Infection of Arabidopsis with *Pseudomonas syringae* pv. *tomato* DC3000 (*Pst*) triggers hypomethylation and transient expression of pericentromeric TEs whose re-silencing by RNA-dependent DNA methylation (RdDM) involves 24 nt small interfering RNAs (siRNAs), which map not only to the TEs but also to distal *PRR/NLR* genes (Pavet et al., 2006; Cambiagno et al., 2018). Furthermore, hypomethylated pericentromeric DNA loci were proposed to control quantitative resistance to *Hyaloperonospora arabidopsidis* (*Hpa*) apparently by regulating defense genes in *trans* (Furci et al., 2019). The control of *NLR* genes by RdDM is not limited to Arabidopsis (Deng et al., 2017; Richard et al., 2018), and besides this control, *PRR/NLR* genes are affected by small RNAs (Yi and Richards, 2007), and miRNA-induced phasiRNA cascades (Liu et al., 2020). As well as DNA methylation, H3K9me2 controls the expression of pathogen defense genes (Dutta et al., 2017), and alternative polyadenylation of particular *NLR* genes (Lai and Eulgem, 2018).

In Arabidopsis, the 5-mC mark is distributed throughout the genome but prevails in TEs concentrated in heterochromatic pericentromeric regions. TE methylation occurs in all sequence contexts (CG, CHG, CHH where H is A, T, or C), whereas gene body methylation mostly targets CG sites. DOMAINS REARRANGED METHYLTRANSFERASE 2 (DRM2) establishes *de novo* methylation at all contexts, guided by siRNAs through the RdDM pathway involving RNA polymerases Pol IV and Pol V (Wendte and Pikaard, 2017). After replication, DNA METHYLTRANSFERASE 1 (MET1), CHROMOMETHYLASE 3 (CMT3), and CMT2/DRM2, maintain DNA methylation at CG, CHG, and CHH sites, respectively. In addition, DNA methylation requires the nucleosome remodeler DEFICIENT IN DNA METHYLATION 1 (DDM1), which provides access to chromatin of epigenetic regulators (Zemach et al., 2013). 5-mC and H3K9me2 act in a positive feedback loop reinforcing heterochromatic TE repression, since the H3K9me2 marks deposited by SUPPRESSOR OF VARIEGATION 3-9 HOMOLOG PROTEIN (SUVH) (Jackson et al., 2004) recruit CMT3 for CHG methylation. In turn, SUVH4 binds to methylated CHG sites (Du et al., 2014) and SUVH9 and H3K9me2 recruit RdDM components (Law et al., 2013; Johnson et al., 2014). Consistently, the *suvh4/5/6* mutants reduce both H3K9me2, and CHG and CHH methylation, and reactivate TE expression (Stroud et al., 2013). On the other hand, the chromatin remodeler MORPHEUS MOLECULE 1 (MOM1) represses a subset of pericentromeric TEs without modifying DNA or histone methylation (Amedeo et al., 2000; Habu et al., 2006; Vaillant et al., 2006). This protein acts on loci methylated by RdDM, and double mutants impaired in MOM1 and RdDM show synergic activation of specific loci (Yokthongwattana et al., 2010). In addition, the effect of these regulators is counteracted by DNA glycosidases from the DEMETER family (Li et al., 2018), and H3K9 demethylase INCREASE IN BONSAI METHYLATION 1 (IBM1) that remove 5-mC and H3K9me2, respectively (Inagaki et al., 2010). Interestingly, some epigenetic marks are mutually exclusive. In Arabidopsis, the histone variant H2A.Z, which is absent from TEs or repeats but present in genes where it modulates nucleosome stability, is anti-correlated with DNA methylation. H2A.Z is frequently associated to the transcriptional start site (TSS), and often marks the body of genes regulated during development or biotic and abiotic stress conditions, under their repressive state (Coleman-Derr and Zilberman, 2012; Berriri et al., 2016). Depletion of H2A.Z impairs disease resistance by perturbing defense genes activation (Berriri et al., 2016). Other epigenetic marks relevant for regulation of defenses against pathogens are H3K4 methylation and histone acetylation. In fact, H3K4me3 is considered a marker of stress memory (Jaskiewicz et al., 2011; Po-Wen et al., 2013; Espinas et al., 2016).

Position and combination of epigenetic marks define distinctive chromatin states, and a given mark is associated with active or repressive functions, depending on its position. Thus, 5-mC is present in promoters of silent genes and TEs, as well as in the coding sequence of constitutively expressed genes (Zhang et al., 2006). H2A.Z strongly correlates with the repressive mark H3K27me3 both at TSS and gene bodies (Carter et al., 2018), and can also be associated to the active H3K4me3 mark at promoters, but in both cases, it negatively correlates with gene expression (Dai et al., 2017). Combination of 16 epigenetic features were initially used to define nine chromatin states correlated with gene expression in the Arabidopsis genome (Sequeira-Mendes et al., 2014). More recently, analysis of 216 epigenomic datasets including histone variants, histone modifications, DNAse treatments (chromatin accessibility), DNA methylation, and transcription factor association, among others, led to the definition of 36 chromatin states in this model (Liu et al., 2018).

Here, we studied the expression of *PRR/NLR* genes and downstream associated defense genes in mutants impaired in deposition of the repressive epigenetic marks 5-mC and H3K9me2 by MET1, CMT3, SUVH4/5/6, or DDM1, and in the negative TE regulator MOM1. Analysis of gene expression, and resistance to *Pst* indicated that all mutants are primed to activate defenses. We found that different *PRR/NLR* gene sets are prone to be induced in the mutants, and different regulators predominantly control specific defense pathways. We provide a description of the epigenetic marks present in promoters and coding sequences of up-regulated genes, showing that not only 5-mC and H3K9me2, but also H3K27me3 and H2A.Z could explain their activation. We discuss how MET1, CMT3, MOM1, SUVH4/5/6, and DDM1 would act in coordination with other regulators to ensure efficient expression of *PRR/NLR* genes avoiding their massive induction.

## Materials and Methods

### Plant Material

Seeds of *Arabidopsis thaliana* accession Columbia (Col-0), *cmt3-11* (CS16392), *drm1-2/drm2-2* (CS16383), *drm1-2/drm2-2/cmt3-11* (CS16384) from the Arabidopsis Biological Resource Center, *mom1-5* (Won et al., 2012), *suvh4/suvh5-2/suvh6-1* (Ebbs and Bender, 2006), and *ddm1-1* (Rangwala and Richards, 2007) plants were used in this study. Seeds were sterilized with 10% (v/v) bleach. After being stratified for 3 days at 4°C, plantlets were germinated on Murashige and Skoog media (Sigma-Aldrich) for 10 days, transferred to soil and then grown under 8 h light/16 h dark cycles at 23°C.

### Plant Infection and Pathogen Growth

Six week old plants were infiltrated with 10^5^cfu/mL (quantification of bacterial content) or 10^7^cfu/mL (gene expression analysis) of *Pseudomonas syringae* pv. *tomato* DC3000 (*Pst*). *Pst* was previously grown on King’s B medium supplemented with kanamycin (50 μg/mL) and rifampicin (100 μg/mL). Bacterial growth was analyzed as previously reported (Cambiagno et al., 2015; Rizzi et al., 2017). Briefly, for each time point and genotype, 12 leaf discs (6-mm diameter) from six infected leaves from three different plants were pooled in two groups (six discs each; technical replicates). Pools of discs were homogenized in 10 mM MgCl_2_ and used to prepare serial dilutions that were plated on King’s B selective medium. The number of colonies forming units (CFU) per area unit (cm^2^) was reported. Values represent mean ± standard deviation (SD) of two technical replicates. Similar results were obtained in two independent infection experiments. Statistical differences were calculated by two-tailed unpaired *t*-test.

### Gene Expression and mRNA-Seq Analysis

**FIGURE 1:**
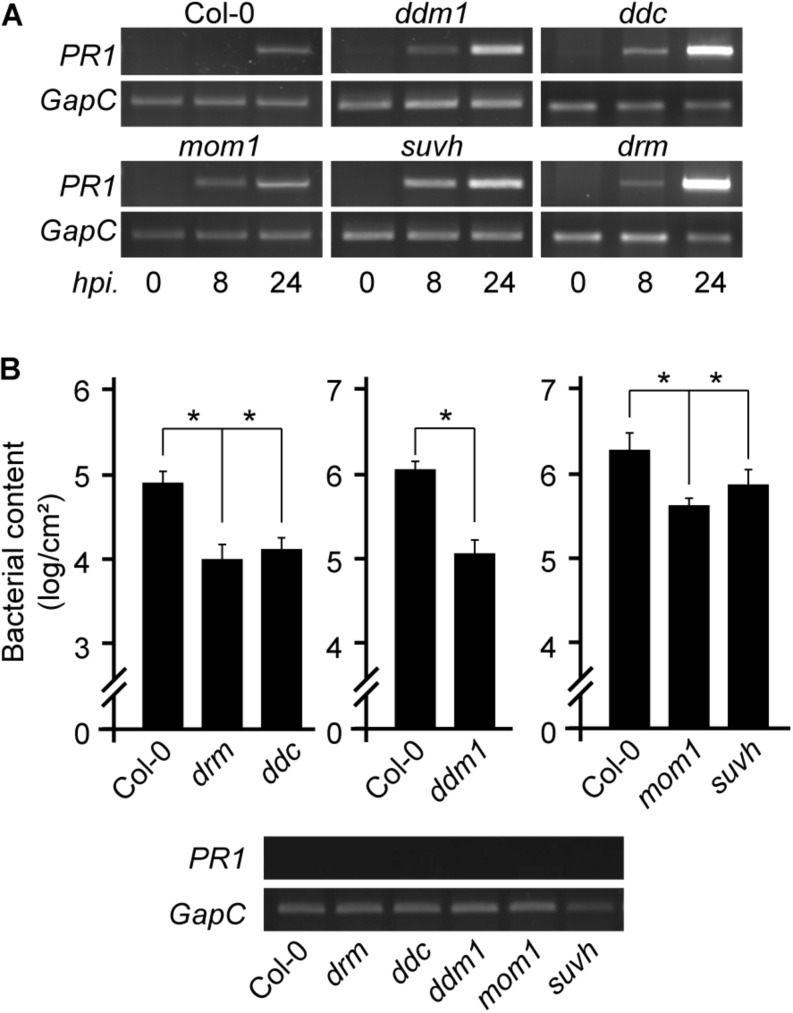
Expression of defense marker gene and resistance to *Pst* infection in chromatin mutant plants. **(A)** *PR1* transcript levels in non-infected or *Pst*-infected plants of the indicated genotypes analyzed at 8 and 24 h post inoculation (hpi). **(B)** *Pst* content in Col-0 wild type and mutant plants at 3 days after infection showing no basal *PR1* expression (bottom). Values represent mean ± standard deviation (SD) of two technical replicates. Similar results were obtained in two independent infection experiments. *p < 0.05 from two-tailed unpaired *t*-test.

**FIGURE 2:**
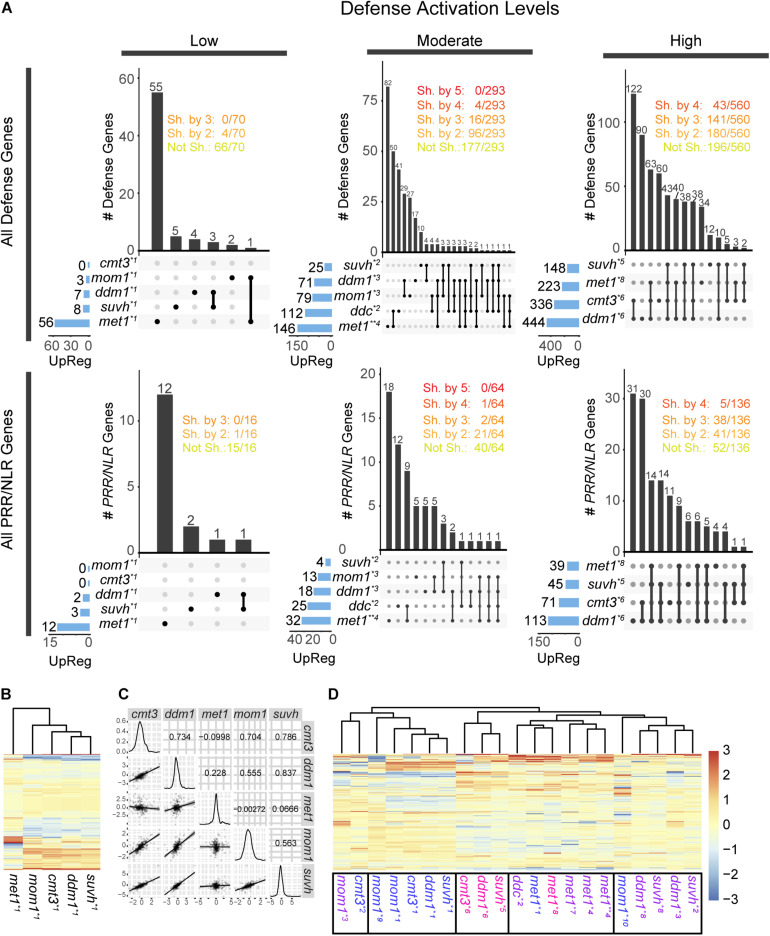
Activation of defense and *PRR*/*NLR* genes in chromatin mutants. **(A)** Intersection of all defense genes (top) or all *PRR/NLR* genes (bottom) activated (FDR < 0.05, FC > 1) in mutant plants. Transcriptome data were analyzed in three groups, corresponding to low, moderate, or high defense activation conditions. The number of activated genes in each sample is described at the left and represented with blue bars. Intersections between mutants are indicated by black lines linking black dots, and the number of genes in the intersection is described above vertical bars. The number of genes shared (Sh. by) or not shared (Not Sh.) among mutants are indicated at the top. **(B)** Clustering of *PRR/NLR* genes (base mean > 10, *n* = 326) in mutant plants under low defense activation conditions. **(C)** Pearson correlation between pair of mutants (top right panels), distribution of FC values in each sample (diagonal), and dot plot and linear regression of pairs of mutants (bottom left) for data shown in **(B)**. **(D)** Clustering of the *PRR/NLR* genes (base mean > 10, *n* = 359) in all the datasets of the different mutants. In **(B,D)**, a Z-score was use to scale the data by mutants (columns). Transcriptomes data analyzed (see Supplementary Table 3): *1: (Stroud et al., 2012), *2:(Stroud et al., 2014), *3:(Bourguet et al., 2018), *4:(Choi et al., 2020), *5:(Zhang C. et al., 2018), *6:(Ning et al., 2020), *7:(Shook and Richards, 2014), *8: (Le et al., 2020), *9:(Moissiard et al., 2014), *10:(Han et al., 2016). Transcriptomes with “low,” “moderate,” or “high” defense activation are indicated with different colors as indicated in Supplementary Table 5.

**FIGURE 3:**
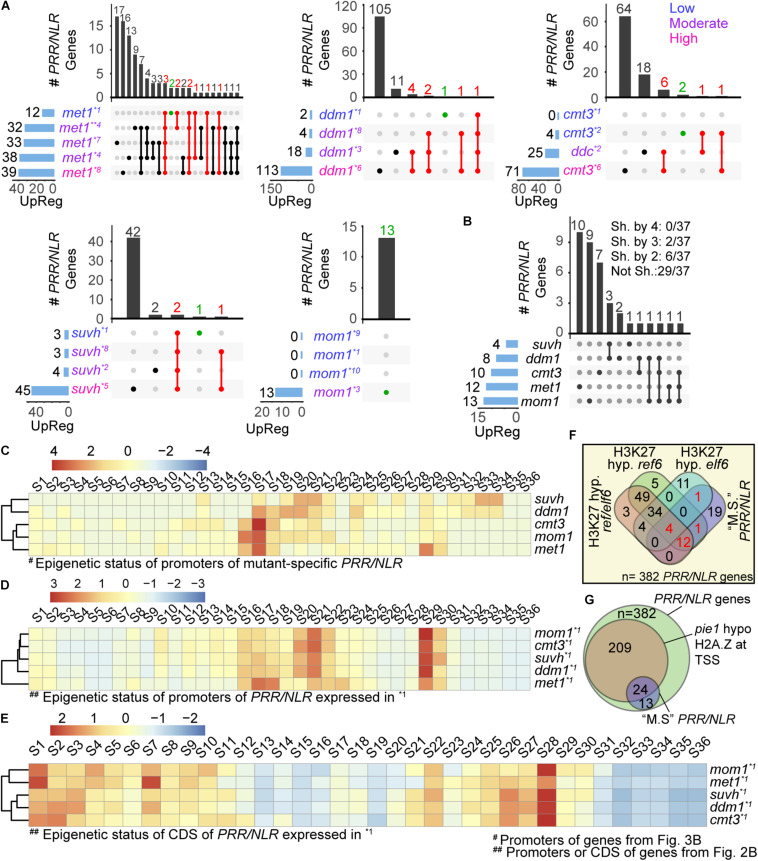
Mutant-specific *PRR/NLR* genes spontaneously activated in each mutant. **(A)** Intersection of *PRR/NLR* genes activated (FDR < 0.05, FC > 1) in each mutant plotted as in Figure 2A. Red intersections and numbers indicate genes that are consistently activated in different transcriptomes (upregulated at “low”/“moderate” -or “low” condition for *met1*- and also at a subsequent condition), and green intersections genes also included in **(B)**. All transcriptome datasets were used to reach at least four mRNA-seq data for each mutant. Transcriptomes with “low,” “moderate,” or “high” defense activation are indicated with different colors. **(B)** Intersection of mutant-specific *PRR/NLR* genes among the different mutants (see section “Materials and Methods”). The number of genes shared (Sh. by) or not shared (Not Sh.) among mutants are indicated at the top. **(C)** Heatmap of the chromatin state of promoters of the 37 *PRR/NLR* genes activated in the mutants **(B)**, described for wild type plants. Clustering is shown in the left. **(D,E)** Heatmap of the chromatin states of promoters **(D)** or coding sequences **(E)** of induced genes shown in Figure 2B (base mean > 10, FC > 0.2, *n* = 193) described for wild type plants. A Z-score was used in all heatmaps to scale the data by mutants (row). **(F)** Venn diagram showing the intersection of 37 mutant-specific *PRR/NLR* from **(B)** (“M.S.” *PRR/NLR*), and the *PRR/NLR* hypermethylated (H3K27 hyp.) in *ref6, elf6, and ref6/elf6.* Red numbers indicate the 18 mutant-specific *PRR/NLR* genes targeted by REF6 and/or ELF6. Fisher’s exact test was applied between mutant-specific *PRR/NLR* genes (this study) and *PRR/NLR* genes hypermethylated in the mutants (Antunez-Sanchez et al., 2020): mutant-specific *PRR/NLR* and H3K27 hyp *ref6*: *p* = 0.011; mutant-specific *PRR/NLR* and H3K27 hyp *erf6/elf6*: *p* = 0.033; mutant-specific *PRR/NLR* and H3K27 hyp *elf6*: *p* = 1. **(G)** Venn diagram showing the intersection of all *PRR*/*NLR* genes, *PRR*/*NLR* genes with decreased amount of H2A.Z in *pie1* and 37 *PRR*/*NLR* genes from **(B)**.

Gene expression was analyzed by RT-sqPCR as described previously (Cambiagno et al., 2015). For each genotype six infected leaves from three different plants were pooled and used for RNA extraction. The experiment was repeated three times with similar results. Total RNA (2 μg) was treated with RQ1 DNAase (Promega) and incubated with random hexamer primers and M-MLV retro-transcriptase (Promega). sqPCR was performed with Taq DNA polymerase (Promega) (3 min at 95°C, 22 cycles of 35 s at 95°C, 35 s at 60°C, and 45 s at 72°C) using primers for *GapC (GADPH C* subunit; *At3g04120;*5′ CACTTGAAGGGTGGTGCCAAG 3′ and 5′ CCTGTTGTCGCCAACGAAGTC 3′) and *PR1* (*At2g14610;* 5′ ATGAATTTTACTGGCTATTCTC 3′ and 5′ AGGGAAGAACAAGAGCAACTA 3′) (Figure 1). mRNA-seq data from at least four transcriptomes of each mutant were evaluated (Supplementary Table 3). The subset of *Pst* mutants used in *Pst* infection assays and transcriptome analysis was not identical because of limitations of publically available data for some particular genotypes. Raw data was obtained from: PRJNA167620, PRJNA222364, PRJEB28655, PRJNA504886, PRJNA437500, PRJNA603988, PRJNA566443, PRJNA238327, DRA009134, PRJNA237334, and PRJNA318519. Trimmed reads were mapped to *Arabidopsis thaliana* genome (TAIR10) with TopHat2 and gene counts were generated with featureCounts (version 1.6.2). Differentially expressed genes between mutants and wild type plants were determined with Deseq2 R package (version 1.20.0) considering a False Discovery Rate (FDR) lower than 0.05 and log2 Fold Change (FC) higher than 1. “Defense Genes” and “*NLR/LRR*” genes have been described in a previous work (Cambiagno et al., 2018). To select the first group we filtered “biotic stress-response genes” from the GO term “Biological process response to biotic or abiotic stimulus.” The *PRR/NLR* group includes *PRR*, *NLR*, *RLK* (receptor-like kinases) and *RLP* (receptor-like proteins) from the mentioned GO term, plus all genes from these categories described by Shiu et al. (2004). Both groups are listed in Supplementary Table 4. Up-regulated genes (FDR < 0.05, FC > 1) intersections between mutants was shown by using UpSet plots (Conway et al., 2017) R package. Heatmaps were performed by using pheatmap library in R (Kolde, 2015). Hierarchical clustering was performed and euclidean distance was calculated between samples. In all cases, data was scaled by column (mutants). All values over −3 and +3 were capped with argument “breaks.” In Figure 2, heatmap and clustering was performed by comparing the FC of *PRR/NLR* genes with a base mean (average of the normalized count values over all samples, being counts corrected for library size by DESeq2) higher than 10. In Figure 3, heat maps and clustering were performed with normalized frequencies of chromatin states found in wild type plants of genes activated (base mean > 10 and FC > 0.2) in different mutants. Chromatin states of different genes were obtained from http://systemsbiology.cau.edu.cn/chromstates/At.php, as was previously described (Liu et al., 2018).

### Chromatin Analysis

The epigenetic marks and chromatin states within promoters (1 kb upstream of coding sequence) or gene bodies for the selected genes were obtained from the site http://systemsbiology.cau.edu.cn/chromstates/ (Liu et al., 2018). Chromatin states were defined within a bin of 200 bp (Liu et al., 2018). The frequency of chromatin states for the genes induced in a mutant was determined as the number of such state normalized by a Z score. Heatmaps showing the normalized frequencies were performed by using pheatmap library in R (Kolde, 2015).

## Results

### Immune Traits of Mutants Impaired in Deposition of Repressive Epigenetic Marks

*Pst* infection triggers dynamic alterations in the Arabidopsis DNA methylome, generating some changes that affect differentially expressed immune receptor genes or TEs linked to them (Dowen et al., 2012; Yu et al., 2013). It is therefore not surprising that mutants like *met1*, carrying abnormal DNA methylation patterns at defense genes, show atypical resistance to pathogens (Dowen et al., 2012; Yu et al., 2013). However, it is curious that *mom1* mutants impaired in constitutive repression of pericentromeric TEs show defense priming against *Pst* (Cambiagno et al., 2018). We here analyzed this trait in other chromatin mutants that lose negative regulation of pericentromeric TEs. We used *drm1/drm2*, *drm1/drm2/cmt3 (ddc)*, *ddm1*, and *suvh4/5/6* plants grown under controlled conditions and without exposure to stress, at early developmental stages, and included *mom1* as control. None of these plants showed constitutive expression of the defense gene marker *PR1* (PATHOGENESIS-RELATED GENE 1) (Figure 1A). However, all mutants showed a faster or stronger *PR1* induction after *Pst* inoculation than wild type plants. These results suggested that the mutants are prone to activate defense genes. Consistently, despite having no basal expression of *PR1*, after being challenged with *Pst* all mutants were able to restrict bacterial growth more efficiently than wild type plants (Figure 1B). This indicated that DRM/CMT3, DDM1, and SUVH4/5/6, as MOM1 (Cambiagno et al., 2018), prevent the plant from acquiring a primed state of defenses against *Pst.*

### Defense Genes That Accompany the Increased Resistance of Mutants

We used publicly available transcriptome data to evaluate the expression of defense genes in uninfected *cmt3*, *mom1*, *suvh4/5/6*, *ddm1, ddc* mutants. We also included *met1*, as a plant known to express defense genes due to changes in their 5 mC marks (Dowen et al., 2012; Yu et al., 2013). mRNA-seq data derived from different types of samples (Supplementary Table 3), such as 10–21 days old plants, plants exposed to continuous light or long day period, or grown in soil or sterile media, but none of them from plants treated with pathogens (Stroud et al., 2012, 2014; Moissiard et al., 2014; Shook and Richards, 2014; Han et al., 2016; Bourguet et al., 2018; Zhang C. et al., 2018; Choi et al., 2020; Le et al., 2020; Ning et al., 2020). We analyzed the expression of all defense genes (1,366, listed in Supplementary Table 4) in each transcriptome, and selected the upregulated genes with adjusted FDR < 0.05 and log2 fold change over 1 (FDR < 0.05, FC > 1; see section “Materials and Methods”). The genes expressed in the mutants are listed in Supplementary Table 5. Next, we distributed the transcriptomes of the same mutant into three different groups based on the criteria that they contained “low,” “moderate,” or “high” amounts of expressed defense genes in this genotype (Supplementary Tables 3, 5). To define categories, we did not use absolute values as thresholds since the mutants contained different maximum number of expressed defense genes. Instead, transcriptomes with the lowest and highest number of expressed genes were included in the “low” and “high” categories, respectively, those differing with these two groups by up to 33% of genes expressed were placed in the same category, and the rest were included in the “moderate” group. In the case of *mom1*, we made an exception and considered the transcriptome with the highest number of activated genes (79) in the moderate group, given that this mutant reaches a lower level of defense activation than the others. Next, we compared the responses of different mutants within each group. At low defense activation condition, samples of *cmt3*, *mom1*, *ddm1*, and *suvh4/5/6* contained up to 8 induced defense genes, whereas that of *met1* had 56 induced genes (Figure 2A). Although several mutants were grown under identical conditions (Stroud et al., 2012) no common genes were detected among all plants and 6% of genes (4/70) were shared between two mutants. The set of induced genes might be associated with genotypes and not environmental conditions, since different mutants were grown under identical conditions (Supplementary Table 3). Similarly, at moderate defense condition (samples from similar growth condition, Supplementary Table 3) only 5.4, 1.3, or 0% of expressed defense genes (16, 4 or 0/293) were common to three, four, or five mutants, respectively. As expected, the number of common activated genes increased in the last group, and 25.1 or 7.6% of genes (141 or 43/560) were shared between three or four mutants, respectively. Curiously, *ddm1* and *suvh4/5/6* shared more genes than other pairs of mutants in the last analysis, since from 148 genes induced in *suvh4/5/6*, 129 were upregulated in *ddm1*. Then, enhanced resistance to *Pst* could derive from activation of different defense gene clusters in each mutant. Consistently, when *PRR/NLR* genes were extracted from the first pool (“all defense genes”) and evaluated independently, we obtained similar results (Figure 2A, bottom).

We compared the response of mutants at low defense activation conditions by performing a clustering analysis based on *PRR/NLR* gene expression at the sample, but not the gene level. To increase the power of analysis and avoid multiple parameters to define the cutoff, we included *PRR/NLR* genes only filtered by an average of the normalized count values over all samples (base mean) > 10 (326 genes; see section “Materials and Methods”) (Figure 2B). Gene expression profiles were similar for *mom1*, *cmt3*, *ddm1* and *suvh4/5/6* mutants but not for *met1* that did not cluster with them (Figure 2B). Consistently, *suvh4/5/6* and *ddm1* showed the highest Pearson correlation values for this set of data, whereas all pairs of mutants that included *met1* had the lowest ones (Figure 2C). Likewise, when we extended the analysis to *PRR/NLR* genes from all available samples (including “moderate” or “high” defense activation; base mean > 10, *n* = 359), *met1* samples were grouped together (Figure 2D), *mom1* and *cmt3* were close to different mutants depending on the dataset, and *suvh4/5/6* and *ddm1* tended to cluster, consistently with previous results (Figure 2A).

### Pattern of Activated *PRR/NLR* Genes in Each Mutant

To explore how mutants activated defenses against pathogens, we analyzed the induction of *PRR*/*NLR* genes (differentially activated genes, filtered as in Figure 2A: FDR < 0.05, FC > 1) under the different conditions studied for each plant. For each genotype, we ordered the transcriptomes according to the number of induced *PRR/NLR* genes (low defense activation condition at the top), and selected genes expressed in more than one condition. Our interest was to evaluate the mutants at conditions emulating early defense induction. Assuming that the number of expressed genes will increase during progression of defense induction, we selected genes upregulated at low/moderate defense conditions that are also induced at a subsequent condition. In *met1* the amount of genes up-regulated in more than one condition was nearly twice than in other mutants. For this reason, we only selected for *met1* genes activated from the low defense stage (shown in red; Figure 3A and Supplementary Table 2). In *met1*, 10 from 12 *PRR/NLR* genes induced at the first condition were also expressed in samples with a higher number of active *PRR/NLRs* genes. A similar pattern was observed for *ddm1*, *cmt3*, and *suvh4/5/6* mutants (Figure 3A). For *mom1*, most datasets showed no *PRR/NLR* genes expressed, but one of them reported the activation of 13 of these genes (Bourguet et al., 2018). Therefore, *met1*, *ddm1*, *cmt3*, and *suvh4/5/6* plants appear to induce specific subsets of *PRR*/*NLR* genes across transcriptomes. Moreover, some specific *PRR*/*NLR* genes would be consistently activated in each mutant although not necessarily expressed in all its transcriptomes (10, 8, 8, and 3 genes shown in red for *met1, ddm1*, *cmt3*, and *suvh4/5/6*, respectively; Figure 3A). Probably, these genes act upstream in the defense pathways, and are regulated by specific epigenetic features that make them prone to be activated.

We wondered whether the *PRR/NLR* genes that are consistently activated in each mutant (red dots in Figure 3A) were those shared among different plants. To test this, we compared this set of genes among mutants, including in the same analysis genes upregulated in the lower defense activation condition (green dots in Figure 3A). We found that none of them were common to all 5 or even 4 mutants (Figure 3B). Furthermore, 78% of expressed *PRR/NLR* genes (29/37) were not shared between mutants, and only a few of them were expressed in two (6/37) or three (2/37) different plants. Therefore, we defined them as “mutant-specific consistently activated” (hereafter “mutant-specific”) *PRR/NLR* genes. The mutants with the highest number of shared genes were *suvh4/5/6* and *ddm1* (3/4 genes induced in *suvh4*/5/6 are also activated in *ddm1*) (Figure 3B). In addition, 10/12 *PRR/NLR* mutant-specific genes in *met1* were specific for this mutant, most of the genes activated in *suvh4*/5/6 were shared with *ddm1*, whereas *mom1* and *cmt3* were partially close to *suvh4/5/6* and *ddm1* (Figure 3B). These results and those described in Figure 2, indicated that different mutants stimulate different master genes.

### Epigenetic Marks of Mutant-Specific *PRR/NLR* Genes

We evaluated the epigenetic status of the 37 mutant-specific *PRR/NLR* genes, considering the 36 different chromatin states defined by Liu et al. (2018). The genes were analyzed in wild type plants under non-infection conditions, where they may harbor repressive marks. The frequency of chromatin states along those *PRR/NLR* genes (normalized with a Z score by sample) was first determined for promoter sequences (1 kb upstream of coding sequence). Accessible chromatin was found in the promoter regions of all 37 *PRR/NLR* genes (states 15–21, 23, 24, and 36; Figure 3C). Clustering analysis of promoter-chromatin states distinguished *suvh4/5/6* and *ddm1* from the other mutants, and revealed that only these plants activated *PRR/NLR* genes carrying 5-mC and H3K9me2 (states 31–34) in wild type plants (Figure 3C). Surprisingly, all mutants were able to activate genes enriched in H3K27me3 and H2A.Z (state S13) marks in wild type plants. In addition, most of them show different combinations of these marks (S2, S8, S10–S15, S25–S28, S31). We further evaluated the overall number of gene promoters, and bins within gene promoters, harboring the epigenetic marks described by Liu et al. (2018) (see section “Materials and Methods”). H2A.Z was the most abundant repressive mark at gene promoters (20/37) followed by H3K27me3 (9/37) (Supplementary Figure 1). Therefore, although genes expressed in the mutants are mostly uncommon, they share two major repressive chromatin states: 5-mC/H3K9me2 (*suvh4/5/6* and *ddm1*) or H3K27me3/H2A.Z (all mutants).

We further evaluated the chromatin states of all induced *PRR/NLR* genes described in Figures 2B,C (base mean > 10, FC > 0.2, *n* = 193), to know whether epigenetic features of promoters or coding sequences correlate with their expression in the mutants. Interestingly, the promoter chromatin states of those genes tend to cluster the mutants together except *met1* (Figure 3D), in a similar manner they clustered when evaluating their downstream gene expression in Figure 2B. Furthermore, this clustering is not conserved when the chromatin states of *PRR/NLR* coding regions were analyzed (Figure 3E). Then, the activation of these mutant-specific *PRR/NLR* genes may depend on the promoter, but not coding region chromatin states, since the latter mostly correspond to active states.

We analyzed whether the mutant-specific *PRR/NLR* genes studied here could be targeted by the H3K27me3 demethylases EARLY FLOWERING 6 (ELF6) and RELATIVE OF EARLY FLOWERING 6 (REF6). Reciprocal crosses of wild type plants and *elf6*/*ref6* double mutants induced epimutations characterized by ectopic accumulation of H3K27me3 in hetero and euchromatin, and showed activation of several biotic stress responsive genes, even when the H3K27me3 demethylase activity was restored in F5 progenies (Antunez-Sanchez et al., 2020). We looked for the presence of *PRR/NLR* genes among those defined as hypermethylated in *ref6/elf6*, *ref6*, or *elf6* mutants (5,414, 5,226, and 1,670 genes, respectively (Antunez-Sanchez et al., 2020). Interestingly, 106, 105, and 54 of the 382 *PRR/NLR* genes analyzed here were detected in *ref6/elf6*, *ref6*, or *elf6*, respectively (Figure 3F). Moreover, 18 genes hypermethylated in these mutants corresponded to mutant-specific *PRR/NLR* genes and 17 of them were present in *ref6*. These 18 genes corresponded to the 33, 12, 50, 53, and 58% of the genes in *ddm1, suvh4/5/6*, *cmt3*, *mom1*, and *met1*, respectively. This indicates that the apparently primed state of some *PRR/NLR* genes analyzed here may be preferentially associated to a *cis*-activity of REF6. Finally, we evaluated the abundance of the H2A.Z histone variant on *PRR/NLR* genes using public data from chromatin immune precipitation studies (Carter et al., 2018). Deposition of this mark mostly depends on the ATPase activity of the SWR1-family chromatin remodeler PHOTOPERIOD INDEPENDENT EARLY FLOWERING1 (PIE1). Interestingly, we found that 24 of the 37 mutant-specific *PRR/NLR* genes have reduced H2A.Z content in *pie1* plants (Figure 3G), suggesting that PIE1-dependent H2A.Z deposition mediates basal repression of these genes.

## Discussion

We here describe that *drm1/drm2*, *ddc*, *ddm1*, and *suvh4/5/6* mutants grown under optimal grown conditions, maintain basal *PR1* repression but restrict *Pst* proliferation more efficiently than wild type plants. These plants lose negative regulation of defense genes under other conditions suggesting that MET1, DRM/CMT3, SUVH4/5/6, and DDM1 would repress defense genes induction. Controlling defense genes through their epigenetic states is advantageous for the plant as it facilitates gene expression without fitness cost (Jaskiewicz et al., 2011; Po-Wen et al., 2013; Espinas et al., 2016). Deposition of active histone marks, such as H3K4me3, H3Ac, and H4Ac, at promoters of pathogen-sensitive WRKY transcription factors or defense genes is known to mediate defense priming (Jaskiewicz et al., 2011; Po-Wen et al., 2013; Espinas et al., 2016), while suppression of H3K4me3 deposition at TSS of defense genes impairs this phenomenon (Mozgova et al., 2015). In addition, H3K9Ac and H3K27me3 are associated to a primed state of defenses in the offspring of infected plants (Luna et al., 2012). Besides this, we found that regulators of H3K9me2 and 5-mC marks could also regulate priming establishment. Lack of MET1, DRM/CMT3, MOM1, SUVH4/5/6, and DDM1 triggers a transcriptional reprogramming of defenses apparently in response to some inadvertent stress or aging. H3K9me2 and 5-mC prevail in heterochromatin, but are present in defense genes activated in the mutants. However, as described later, other induced genes do not carry these marks suggesting they are regulated by more complex mechanisms.

Our results could suggest that defense priming can be controlled upstream of the immune cascades by activating *PRR*/*NLR* genes. Consistently, a recent study analyzed priming-associated open chromatin sites and suggested immune receptor genes as new markers for SAR in Arabidopsis (Baum et al., 2019). Our deep sequencing data analysis showed that *suvh4/5/6*, *ddm1, cmt3*, and *met1* mutants lose basal repression of *PRR/NLR* genes in at least one of the conditions analyzed. These genes have no constitutive expression in the mutants, but are expressed under some conditions suggesting they may be prone to be induced in these plants. Some of these genes (37) were expressed at low defense activation conditions, when other defense genes are induced but *PR1* expression was not yet evident, recapitulating the phenotypes described for *mom1* (Cambiagno et al., 2018). The finding that none of these 37 genes are shared between the five genotypes, and that 29 of them are unique to a single mutant indicated that each mutant stimulates a particular defense pathway. However, common branches of the defense pathways could be activated in *suvh4/5/6* and *ddm1*, which share three induced immune receptor genes. The ability to control different *PRR/NLR* genes through different regulators could represent an adaptive advantage since it would prevent a massive induction when a particular regulator fails. Interestingly, the pattern of *PRR/NLR* expression is not stochastic as the same group of genes is expressed under different conditions in each mutant. Moreover, an original nucleus of genes is conserved when the number of induced defense genes increases suggesting that the mutant-specific *PRR/NLR* genes can act as hubs for subsequent defense activation. Even so, it is expected that defense priming by epigenetic regulation of *PRR/NLR* genes will coexist with up-regulation of downstream genes (Jaskiewicz et al., 2011; Luna et al., 2012; Po-Wen et al., 2013; Baum et al., 2019).

The analysis of chromatin status of mutant-specific *PRR/NLR* genes showed some curious results. Traits of promoters, but not coding sequences (Figures 3D,E), correlated with mutant clustering by *PRR/NLR* expression (Figure 2B), suggesting that promoter marks regulated gene activity. As expected, accessible chromatin traits prevailed in these promoters (Liu et al., 2018). Moreover, 15 genes belonged to the group of 53 *PRR*/*NLR* genes described by Baum et al. (2019) as showing both increased chromatin accessibility after SAR induction and higher expression after challenge of systemic tissues. Promoters of some of the *PRR/NLR* genes in *suvh4/5/6* and *ddm1* carried 5-mC and H3K9me2 in wild type plants, suggesting they may be controlled in *cis* by SUVH4/5/6 and DDM1. These genes may also be affected by priming or aging since many of them do not show constitutive expression in *suvh4/5/6* and *ddm1* (i.e., not expressed in ddm1^*1^ or suvh4/5/6^*1^ transcriptomes), as described for other *PRR/NLR* genes misregulated in MOM1 (Cambiagno et al., 2018). Curiously, other genes activated in the mutants contained unexpected marks. This was the case of those induced in *met1* and *cmt3*, which did not contain 5-mC, but H3K27me3/H2A.Z, in wild type plants. The presence of H3K27me3 and H2A.Z at promoters of *PRR/NLR* genes expressed in *suvh4/5/6*, *ddm1* and *mom1* was also unexpected, and suggested a conserved regulatory effect of these marks on them. H3K27me3 and H2A.Z are antagonistic to DNA methylation (Qiu et al., 2019; Antunez-Sanchez et al., 2020), which is deposited by CMT3 and MET1 and potentiated by SUVH4/5/6 (Zhang H. et al., 2018). H2A.Z was found necessary for basal and effector-triggered resistance and proper expression of defense genes (Berriri et al., 2016). To our knowledge, no previous study reported the involvement of H2A.Z in repression of priming-target genes. Our results suggest that H2A.Z could play a central role in *PRR/NLR* gene priming. Besides this, we noticed that some *PRR/NLR* genes expressed in all our mutants were included among those regulated by the H3K27me3 demethylase REF6 (Antunez-Sanchez et al., 2020). This reinforces the notion that several epigenetic marks maintain different sets of *PRR/NLR* genes in a repressed state but suitable for their rapid induction. Eventually, activation of *PRR/NLR* genes would initially result from H3K27me3/H2A.Z rather than H3K9me2/5-mC remodeling, and the subset of induced genes would depend on the combination of H3K27me3/H2A.Z changes with the activity of each different regulator. However, further studies will be required to determine the effects of MOM1, SUVH4/5/6, DDM1, MET1, or CMT3 deficiency on the H2A.Z or H3K27me3 marks present in these *PRR/NLR* genes.

It is worth noting that all the epigenetic regulators analyzed here maintain repression of pericentromeric heterochromatic TEs and that several studies describe that pericentromeric heterochromatin relaxation enhances biotic stress responses by affecting defense genes in *trans*. MOM1 and siRNA mediate co-regulation of pericentromeric TEs and distal *PRR/NLR* genes (Cambiagno et al., 2018). Epimutant lines obtained by crossing *ddm1* with wild type plants are primed to counteract *Hpa* infection activating defense genes whose methylation is *ddm1*-independent (Furci et al., 2019). A subset of defense genes mediating *Hpa* resistance is antagonistically regulated by NRPE1 and ROS1, but none of these genes are target of DNA methylation (Lopez Sanchez et al., 2016). Changes in the H3K27me3 marks of *ref6/elf6* epimutants impact the pericentromeric regions and the expression of defense and *PRR/NLR* genes (Antunez-Sanchez et al., 2020) without altering their H3K27me3 content. Therefore, we cannot rule out that some of the *PRR/NLR* genes de-repressed in the mutants respond to this type of mechanism.

In summary, we showed that complex interactions between chromatin and epigenetic remodelers collectively regulate defense priming against pathogens activated by particular *PRR/NLR* genes. It is still unknown how these epigenetic remodelers act together over these genes. Our results and those discussed here, suggest that defense priming not only requires a *cis* effect of the removal of repressive histone marks on defense gene promoters, but may be also sensitive to relaxation and loss of repressive marks from pericentromeres and/or distal euchromatic domains containing TEs, with siRNA-dependent silencing modulating defense genes in *trans*. Additional studies will be required to determine the epigenetic pattern of *PRR/NLR* genes in the mutants here analyzed, and understand how regulators collectively control their expression.

## Data Availability Statement

The original contributions presented in the study are included in the article/Supplementary Material, further inquiries can be directed to the corresponding author/s.

## Author Contributions

DC and MA: conceptualization and writing, founding acquisition. DC and JT: experiments. DC: data analysis. MA: background, supervision. All authors contributed to the article and approved the submitted version.

## Conflict of Interest

The authors declare that the research was conducted in the absence of any commercial or financial relationships that could be construed as a potential conflict of interest.

## Publisher’s Note

All claims expressed in this article are solely those of the authors and do not necessarily represent those of their affiliated organizations, or those of the publisher, the editors and the reviewers. Any product that may be evaluated in this article, or claim that may be made by its manufacturer, is not guaranteed or endorsed by the publisher.

## Supplementary Material

Supplementary Figure 1 Epigenetic marks within the 37 *PRR/NLR* genes shown in Figure 3B. Bar plot showing the number of promoters, or bins (200 bp) within promoters harboring each epigenetic mark described in all the 36 chromatin states from Liu et al. (2018). Repressive marks are indicated in red.

Supplementary Table 1 Fold change of defense genes in the mutants here analyzed, according to data described in legend of Figure 2.

Supplementary Table 2 Mutant-specific *PRR/NLR* genes induced in the mutants here analyzed.

Supplementary Table 3 Transcriptomes analyzed in this study.

Supplementary Table 4 Defense genes and *PRR/NLR* genes analyzed in this study.

Supplementary Table 5 Defense genes and *PRR/NLR* genes up-regulated in *met1*, *ddm1*, *cmt3*, *suvh4/5/6*, and *mom1* mutants.
