# Supplementary figures and images for: Convergent Epigenetic Mechanisms Avoid Constitutive Expression of Immune Receptor Gene Subsets
Source: Front Plant Sci. 2021 Sep 7;12:703667. doi: 10.3389/fpls.2021.703667 (PMC8452986; doi:10.3389/fpls.2021.703667)

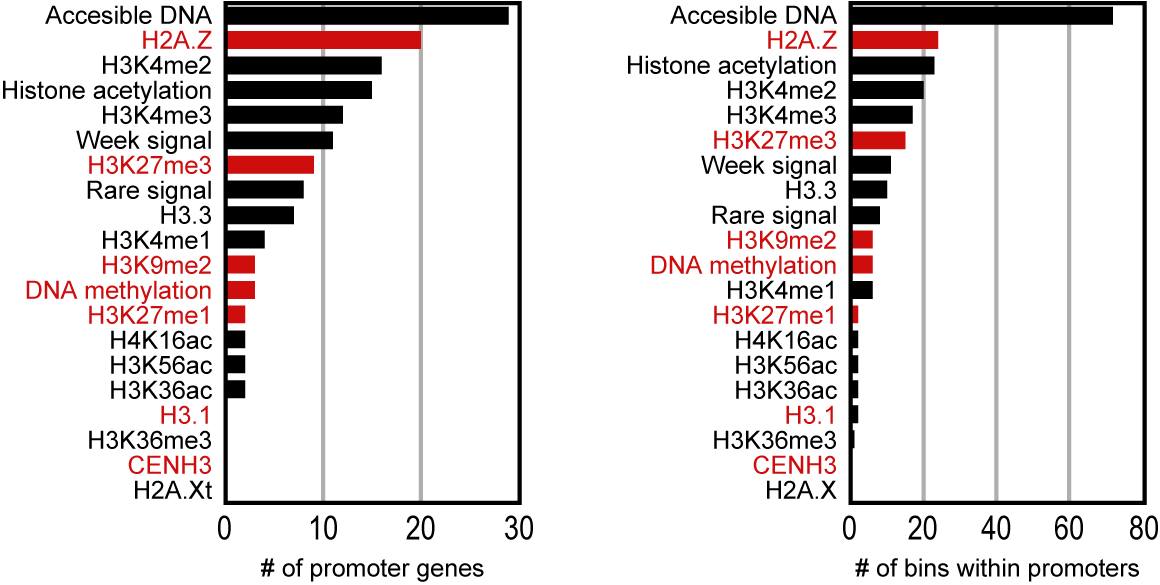

Supplement: Supplementary Figure 1 — Epigenetic marks within the 37 PRR/NLR genes shown in Figure 3B. Bar plot showing the number of promoters, or bins (200 bp) within promoters harboring each epigenetic mark described in all the 36 chromatin states from Liu et al. (2018). Repressive marks are indicated in red. [file Image_1.tif]
